# The role of low-carbohydrate, high-fat diet in modulating autophagy and endoplasmic reticulum stress in aortic endothelial dysfunction of metabolic syndrome animal model

**DOI:** 10.3389/fnut.2024.1467719

**Published:** 2024-11-13

**Authors:** Basmah Eldakhakhny, Abdulhadi Bima, Aliaa A. Alamoudi, Abrar Alnami, Salwa Mohamed Abo-Elkhair, Hussein Sakr, Yousef Almoghrabi, Fatma Mohamed Ghoneim, Reham Mohamed Nagib, Ayman Elsamanoudy

**Affiliations:** ^1^Department of Clinical Biochemistry, Faculty of Medicine, King Abdulaziz University, Jeddah, Saudi Arabia; ^2^Food, Nutrition, and Lifestyle Research Unit, King Fahd for Medical Research Centre, King Abdulaziz University, Jeddah, Saudi Arabia; ^3^Regenerative Medicine Unit, King Fahd Medical Research Center, King Abdulaziz University, Jeddah, Saudi Arabia; ^4^Department of Medical Biochemistry and Molecular Biology, Faculty of Medicine, Mansoura University, Mansoura, Egypt; ^5^Department of Physiology, College of Medicine and Health Sciences, Sultan Qaboos University, Muscat, Oman; ^6^Department of Medical Physiology, Faculty of Medicine, Mansoura University, Mansoura, Egypt; ^7^MBBS Program, Department of Physiological Sciences, Fakeeh College for Medical Sciences, Jeddah, Saudi Arabia; ^8^Department of Medical Histology and Cell Biology, Faculty of Medicine, Mansoura University, Mansoura, Egypt; ^9^Department of Anatomical Pathology, Faculty of Medicine, Mansoura University, Mansoura, Egypt

**Keywords:** metabolic syndrome, endothelial dysfunction, oxidative stress, endoplasmic reticulum stress, autophagy, low-carbohydrate, high-fat diet

## Abstract

**Background:**

Endothelial dysfunction (ED) is induced by insulin resistance, mediated by endoplasmic reticulum (ER) stress and disturbed autophagy. This study investigates the protective role of a low-carbohydrate, high-fat (LCHF) diet on ED, ER stress, and autophagy dysregulation in an experimental animal model of metabolic syndrome.

**Methods:**

Forty male Sprague–Dawley rats were divided into four groups: a Control group (standard diet) and three Dexamethasone (DEX) treated groups. Group II continued the standard diet, Group III received an LCHF diet, and Group IV received a high-carbohydrate, low-fat (HCLF) diet. At the end of the experiment, aortic tissue samples were obtained and used for histological, immunohistochemical (Endothelin and PCNA, biochemical MDA, TCA, NO, 8-OH-dG, and Nrf2/ARE protein) and molecular (Endothelin, eNOS, Nrf-2 *α*, p62, LC3, BECN-1, PINK1, CHOP, BNIP3, PCNA) analysis.

**Results:**

Oxidative stress, autophagy markers, and ED markers are increased in the metabolic syndrome group. LCHF diet mitigates the adverse effects of DEX on endothelial dysfunction and oxidative stress, as evidenced by reduced BMI, HOMA-IR, and improved histological and molecular parameters.

**Conclusion:**

Oxidative stress, autophagy dysregulation, and ER stress play crucial roles in the pathogenesis of insulin resistance-induced endothelial dysfunction. An LCHF diet offers protective benefits against insulin resistance and related comorbidities, including endothelial dysfunction.

## 1 Introduction

Insulin resistance refers to the decreased responsiveness of tissues that target insulin, even at elevated physiological levels of the hormone. It is considered the pathogenic driver of many modern diseases, including metabolic syndrome, nonalcoholic fatty liver disease (NAFLD), atherosclerosis, and type 2 diabetes (T2DM). In the pre-diabetic condition, insulin levels increase to compensate for insulin requirements, leading to chronic hyperinsulinemia, hyperglycemia-induced *β*-cell failure, and eventually T2DM (1). Notably, the glucose-regulating effects of insulin, such as the suppression of hepatic glucose production (HGP) and lipolysis, cellular uptake of plasma glucose, and net glycogen synthesis, are not observed in insulin-resistant tissues at normal physiological levels (2).

The endothelium, a specialized and extensive tissue in the body, regulates the normal function of blood vessels by acting as a mechanical lining. It also plays a pivotal role in regulating leucocyte adhesion, platelet aggravation, and blood vessel patency and controls the release of secretory factors in response to mechanical stimuli (3). The major role of endothelium is to ensure adequate blood flow, which depends on the counterbalance between vasodilators and vasoconstrictors. Vasodilators, including prostacyclin (PGI2) and nitric oxide (NO), aim to maintain adequate blood by dilating the vessels, while vasoconstrictors, including endothelin-1 (ET1) and thromboxane A2 (TXA2), counterbalance the excessive vasodilation and maintain the vascular tone (4).

Different factors may lead to endothelial dysfunction, including insulin resistance and diabetes (5). One significant pathological mechanism underlying endothelial dysfunction in diabetes mellitus and its related insulin resistance states is endoplasmic reticular (ER) stress (6). The endoplasmic reticulum (ER) is an essential site for many cellular functions, including post-translational folding and synthesis of secretory and transmembrane proteins in eukaryotic cells. ER stress occurs under various physiological or pathophysiological conditions that increase protein demand or accumulate misfolded and/or unfolded proteins within the ER lumen. The unfolded protein response (UPR) is an adaptive signaling cascade activated by ER stress to reduce protein synthesis. It aims to enhance protein folding capacity and degrade irreversibly misfolded proteins, restoring ER homeostasis (7). However, the chronic activation of UPR leads to a state of ER stress and the activation of pro-apoptotic signals (8), subsequently leading to apoptosis and inflammation, contributing thus to the progression of cardiovascular disease (7, 8).

Autophagy (self-eating), which is vital for the clearance of toxic proteins and re-cycling of cytosolic content, was found to be activated as a protective mechanism to decrease unfolded protein load and prevent ER-stress-mediated apoptosis (9). Autophagy has been linked to the three signaling arms of UPR (10). For example, activating transcription factor 4 (ATF-4), a key player in UPR, enhances the transcription of autophagy genes: microtubule-associated protein-1 light chain 3 (MAP1LC3B), Beclin-1 (BECN1), autophagy-related 3 (ATG3) and autophagy-related 12 (ATG12). Additionally, cleaved ATF-6, another important UPR transcription factor, increases the expression of death-associated protein kinase 1 (DAPK1), phosphorylating BECN1, leading to autophagy. Moreover, activating IRE-1α triggers MAPK, ultimately resulting in the cessation of autophagy activation (7, 11).

The relationship between ER stress and insulin signaling disruption is complex and interdependent. On the one hand, ER stress is known to be enhanced by obesity and has been proposed to induce insulin resistance in liver and pancreatic *β*-cells (12). On the other hand, during an over-nourished condition, the liver responds by enhancing UPR by accumulating unfolded proteins in the ER. During this process, the recruitment of glucose-regulated protein 78 (GRP78, also known as BiP) results in the activations of all three signaling arms of UPR and, thus, reduces unfolded protein levels (13). Experimentally, ER stress induction suppresses insulin signaling via the serine phosphorylation of insulin receptor substrate-1 (IRS-1) by c-Jun N-terminal kinase (JNK), thus interfering with IRS-1 function in insulin signaling. As in the liver, increased demand for insulin secretion induces ER stress in pancreatic *β*-cells in chronic hyperglycemic diabetic humans and mice, contributing to the development of T2DM. Pancreatic *β*-cell-specific knockout of X-box-binding protein-1 (XBP-1), a key transcription factor in ER stress, resulted in hyperglycemia and diet-induced insulin resistance from β-cell dysfunction in mice. Thus, while it is premature to conclude that ER stress directly induces insulin resistance and vice versa, ER stress seems to regulate glucose and lipid metabolism, such as lipogenesis, lipid droplet formation, and lipid storage (1). Thus, dietary composition would be expected to influence metabolic pathways and cellular stress responses significantly.

Limiting carbohydrate intake has a long-standing history, once serving as a treatment for type 1 diabetes (T1D) before the discovery of insulin (14). Reducing ER stress and its associated pathological effects is a key mechanism supporting the role of a low-carbohydrate diet (LCD) in preventing comorbidities related to insulin resistance (15).

Accordingly, the current study aims to investigate the potential protective role of a low-carbohydrate, high-fat (LCHF) diet against developing endothelial dysfunction, endoplasmic reticulum stress, and autophagy dysregulation in an experimental animal model of drug-induced metabolic syndrome. Understanding these relationships may reveal novel dietary strategies to mitigate ER stress-related metabolic disorders.

## 2 Materials and methods

This study is a part of an extensive project previously outlined by Alnami et al. (16). The Biomedical Ethics Research Committee has been approved at the Faculty of Medicine, King Abdulaziz University in Jeddah, Saudi Arabia, with reference number 33–21. Additionally, it received authorization from the Animal Care and Use Committee (ACUC) at the King Fahd Medical Research Center, affiliated with King Abdulaziz University in Jeddah, under the protocol number ACUC-20-09-21.

### 2.1 Animals and experimental protocol

Forty male Sprague–Dawley rats, weighing around 120 ± 20 grams, were acquired from the Animal House at King Fahd Medical Research Center, Jeddah, Saudi Arabia. Initially, the animals were acclimatized for 10 days in a controlled environment and maintained until they reached 8 weeks of age. They were then divided into four groups of 10 rats each. The first group (Control) was given a standard diet and subcutaneously injected with saline. The remaining groups were treated with Dexamethasone (DEX) (Saudi Pharmaceutical Industries (SPI), Riyadh, Saudi Arabia) with daily subcutaneous injection (250 μg/kg) for 4 weeks. Group II continued the standard diet, Group III received a low-carbohydrate, high-fat (LCHF) diet, and Group IV received a high-carbohydrate, low-fat (HCLF) diet. All diets were isocaloric. The specific details of the diet compositions and methodologies have been extensively detailed in previous studies by Alnami et al. (16) and Bima et al. (17). Body Mass Index (BMI) was calculated based on the weekly height and body weight measurements.

### 2.2 Sampling

By the end of the four-week experiment, the rats were anesthetized by ether after 12 h of overnight fasting. Then, the heart was exposed via a thoracic incision. Intracardiac perfusion using normal saline followed by 10% neutral-buffered formalin for the partial fixation of the specimens was conducted.

The descending aorta was meticulously dissected, and blood, connective tissue, and fat were removed. Each vessel was cut into 3–4 mm rings, weighed, and homogenized for biochemical analysis. For homogenization, the aorta vessels were immediately collected and rinsed with ice-cold PBS (pH 7.4). Then, homogenization was carried out in 0.1 M phosphate buffer, pH 7.4, containing 0.15 M KCl, 0.1 mM EDTA, 1 mM DTT, and 0.1 mM phenylmethylsulfonyl fluoride at 4°C. The samples were centrifuged at 14,000 rpm for 20 min at 4°C, and protein content was determined using the Bradford method (18).

About 20–40 mg tissue samples from the cerebral cortex and carotid artery were snap-frozen in liquid nitrogen and stored at −80°C for molecular investigations for total RNA extraction. Another aorta samples were subjected to paraffin fixation and used for histological examination.

### 2.3 Histological study

Specimens from the aorta were fixed in Bouin’s solution. After fixation, specimens were dehydrated in an ascending series of alcohol, cleared in two changes of xylene, and embedded in molten paraffin. Sections of 5 microns thickness were cut using a rotary microtome and mounted on clean slides. For histological examination, sections were stained with hematoxylin and eosin (H&E), according to Bancroft and Layton (19).

### 2.4 Immunohistochemical (IHC) study

For immunohistochemical detection of endothelin and proliferating cell nuclear antigen (PCNA) reactions, sections from the aorta were taken on positive slides and immunostained using a standard avidin biotin peroxidase complex system. Endothelin staining was performed using specific antibodies (Pierce #MA3-005: 1:200; Abcam #117757: 1:450) (20), and PCNA staining was performed using an anti-PCNA mouse monoclonal antibody (cat. no. ab29; 1:200; Abcam, Cambridge, MA, USA) (21) followed by diaminobenzidine visualization (22). As a negative control, PBS replaced the primary antibody. A brown staining in the cytoplasm (endothelin) and nucleus (PCNA) indicates a positive reaction.

### 2.5 Morphometric study

The area percentage of endothelin and PCNA immunoreactions was measured using five immune stained slides of five different rats for each group. Images of immunostained cells were transferred for analysis to an Intel Core I3-based computer using VideoTest Morphology software (Saint Petersburg, Russia) with a built-in routine for area percent measurement.

### 2.6 Biochemical investigations

Aortic tissue homogenate content of Malondialdehyde (MDA) and total antioxidant capacity (TCA) were measured in nmol/g tissue and ng/mg protein, respectively (23) using the colorimetric kits with catalog numbers (cat. no. MD 2528) for MDA and (cat. no. # TA 25 12) for TCA. Bio-Diagnostics, Dokki, Giza, Egypt supplied both kits. Furthermore, the oxidative stress index (OSI) was calculated as follows: OSI (arbitrary unit) = MDA /TAC X 100 (24).

The nitric oxide (NO) level in the tissue homogenate was also estimated by the colorimetric method (Nitric Oxide Assay Kit, Abcam Co., Boston, MA, USA, ab272517), and the results were expressed as μmol/L Ghoneim et al. (94). According to the manufacturer’s instructions, the aortic homogenate concentration of 8-OH-dG was quantified using an ELISA kit (FineTest, Rat 8-OHdG (8-Hydroxydeoxyguanosine)- ELISA Kit, Catalog no.: ER1487-HS) (18).

The expression of the Nrf2/ARE protein was estimated using An Enzyme-Linked Immunosorbent assay (Abcam, Nrf2 Transcription Factor Assay Kit; Catalog No.: ab207223, UK). The procedure followed the manufacturer’s guidelines, and absorbance was measured with an ELISA plate reader at 450 nm, using 665 nm as a reference wavelength.

### 2.7 Molecular studies

Protocol for RNA extraction was described previously (17, 25). The “rats” primer sequences used are presented in Table 1. GAPDH was used as an internal control to normalize the expression of the analyzed genes.

**Table 1:** The primer sequences of the analyzed genes.

Gene		The primer sequence	Reference
Endothelin	Forward	5′- CAACCAGACACCGTCCT CTT-3’	(91)
	Reverse	5′- CTTGGAAAGCCACAAACAGC-3’	
eNOS	Forward	5′- ACCGCCACACAGTAAATCCA-3′	(25)
	Reverse	5′- TGCCAACAGGAAGCTGAGAG-3′	
Nrf-2 α	Forward	5′- CACATC CAG ACA GAC ACC AGT-3’	(92)
	Reverse	5′- CTA CAA ATG GGA ATG TCT CTG C -3’	
p62	Forward	5′-TCCCTGTCAAGCAGTATCC-3′	(93)
	Reverse	5′-TCCTCCTTGGCTTTGTCTC-3′	
LC3	Forward	5′-CCTGCTGCTGGCCGTAGT-3′	(94)
	Reverse	5′- TGATGAAGTCTTCCTGCCAAAA-3′	
BECN-1	Forward	5′- AGCACGCCATGTATAGCAAAGA −3′	(94)
	Reverse	5′- GGAA-GAGGGAAAGGACAGCAT −3`	
PINK1	Forward	5′- CCC ACA CCC TAA CAT CAT CC -3`	(95)
	Reverse	5′- CTG CTC CTC AAG GTA CTG GC -3’	
CHOP	Forward	5′- GAAAGCAGAAACCGGTCCAAT −3′	(94)
	Reverse	5′- GGATGAGATATAGGTGCCCCC -3′	
BNIP3	Forward	5′- TCTGGACGAAGCAGCTCCAA −3′	(96)
	Reverse	5′- CCAAAGCTGTGGGTGTCTATTTCA −3′	
PCNA	Forward	5′- CTCACGTCTCCTTAGTGCAGCTT-3′	(97)
	Reverse	5′- CGATCGCAGCGGTATGTGT −3′	
GAPDH	Forward	5′- TCC CTC AAG ATT GTC AGC AA −3’	(98)
	Reverse	5′- AGA TCC ACA ACG GAT ACA TT −3’	

### 2.8 Statistical analysis

Data analysis was conducted using GraphPad Prism version 10.2.1. A one-way ANOVA followed by Tukey’s post-hoc test was employed to compare quantitative parametric data. The Pearson correlation coefficient (r) assessed linear relationships between parameters. Results are presented as the mean ± standard error of the mean (SEM), with a *p*-value below 0.05 considered statistically significant.

## 3 Results

### 3.1 Effect of LCHF diet on the anthropometric parameters and HOMA-IR

This study is part of a large project, and anthropometric parameters and HOMA-IR were previously described by Alnami et al. (16) and Bima et al. (17). In Summary, the Dexamethasone group (DEX group) exhibited a marked increase in BMI and HOMA-IR levels, confirming the in-duction of metabolic syndrome traits (*p* < 0.0001 and *p* < 0.001, respectively). Conversely, the DEX + LCHF diet significantly mitigated these increases, highlighting its protective potential against insulin resistance and metabolic syndrome (*p* < 0.01 for BMI reduction; *p* < 0.001 for HOMA-IR decrease). The DEX + HCLF group saw an exacerbation of these markers, further emphasizing the detrimental metabolic effects of a high-carbohydrate, low-fat diet (p < 0.01 for BMI increase; p < 0.001 for HOMA-IR elevation).

### 3.2 Effect of LCHF diet on endothelial dysfunction and oxidative stress

**Figure 1:**
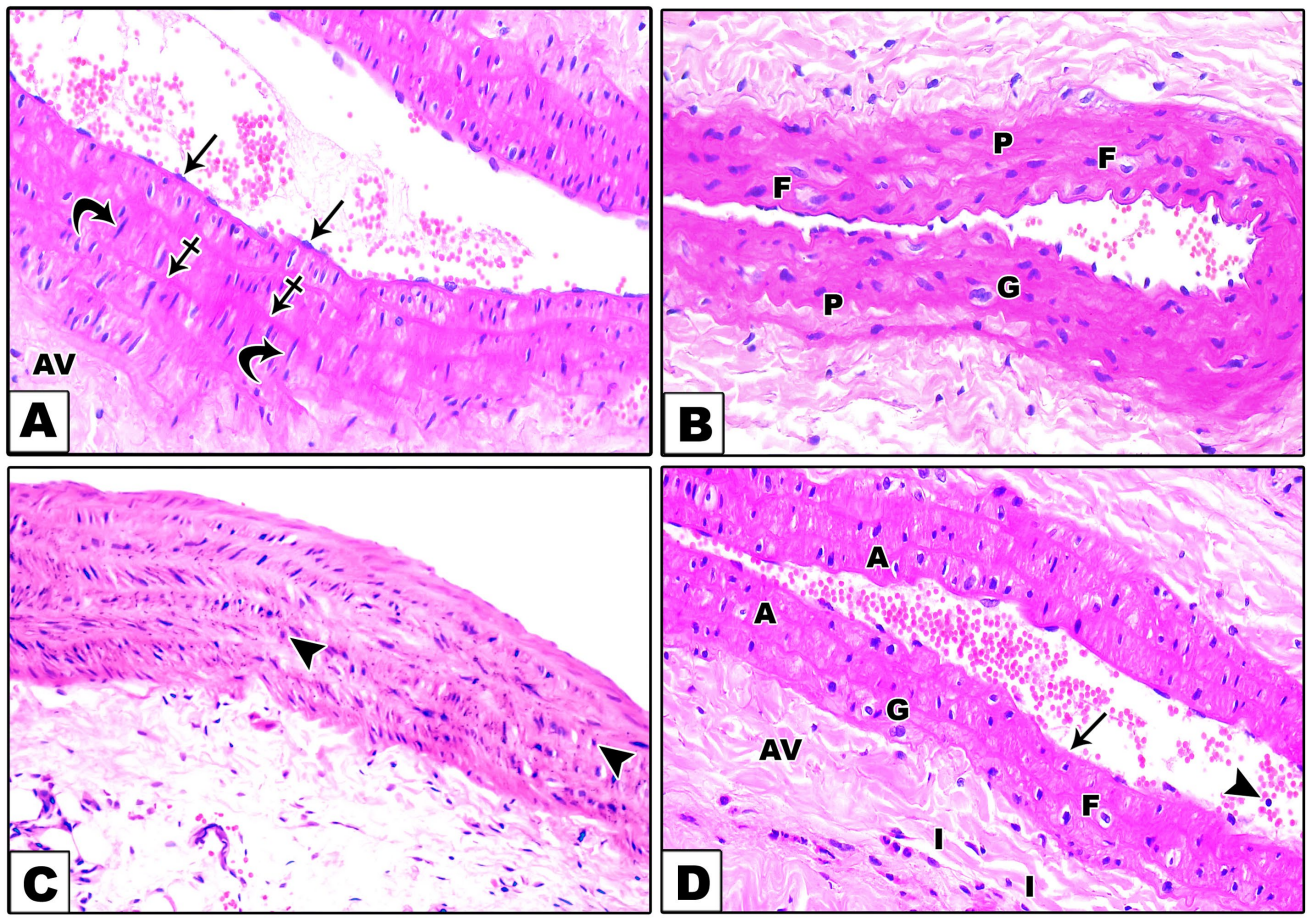
A Photomicrograph of sections in the aorta stained with H&E. (A). In the control group, the wall of the aorta consists of very thin tunica intima lined by endothelial cells (arrows), tunica media which is formed of wavy elastic fibers (crossed arrows), and smooth muscle fibers (curved arrows) with large oval nuclei and tunica adventitia (AV) which is composed of slightly dense connective tissue. (B). In the DEX-treated group, there are giant multinucleated cells (G), foam cells (F), and plump nuclei (P) of some smooth muscle fibers of the tunica media. (C). In the DEX + LCHF treated group, the aorta is improved apart from round nuclei (arrowheads) of some tunica media smooth muscle fibers. (D). In the DEX + HCLF treated group, some endothelial cells are lost (arrow), and others are shed into the lumen (arrowheads) of the aorta. In the tunica media, there is the disorientation of the smooth muscle cells with the presence of giant multinucleated cells (G), foam cells (F), and apoptotic cells (A) with dark pyknotic nuclei. Inflammatory cells (I) are seen in the tunica adventitia (AV).

Hematoxylin and eosin (H&E) staining revealed distinct histological features across the different groups. In the control group, the aortic structure showed typical features, with the tunica intima, tunica media, and tunica adventitia clearly demarcated. The tunica intima was lined with endothelial cells, the media was composed of smooth muscle fibers with wavy elastic fibers, and the adventitia featured slightly dense connective tissue (Figure 1A). In contrast, the DEX group showed histological alterations with the presence of giant multinucleated cells and foam cells, along with plump nuclei of some smooth muscle fibers of the tunica media indicative of vascular inflammation and atherosclerosis (Figure 1B). The DEX + LCHF group displayed a restoration toward normal aortic morphology, albeit with the presence of round nuclei in some smooth muscle fibers of the tunica media (Figure 1C). DEX + HCLF Group exhibited significant degenerative changes, including endothelial cell loss, smooth muscle cell distortion, and inflammatory cells in the tunica adventitia (Figure 1D).

**Figure 2:**
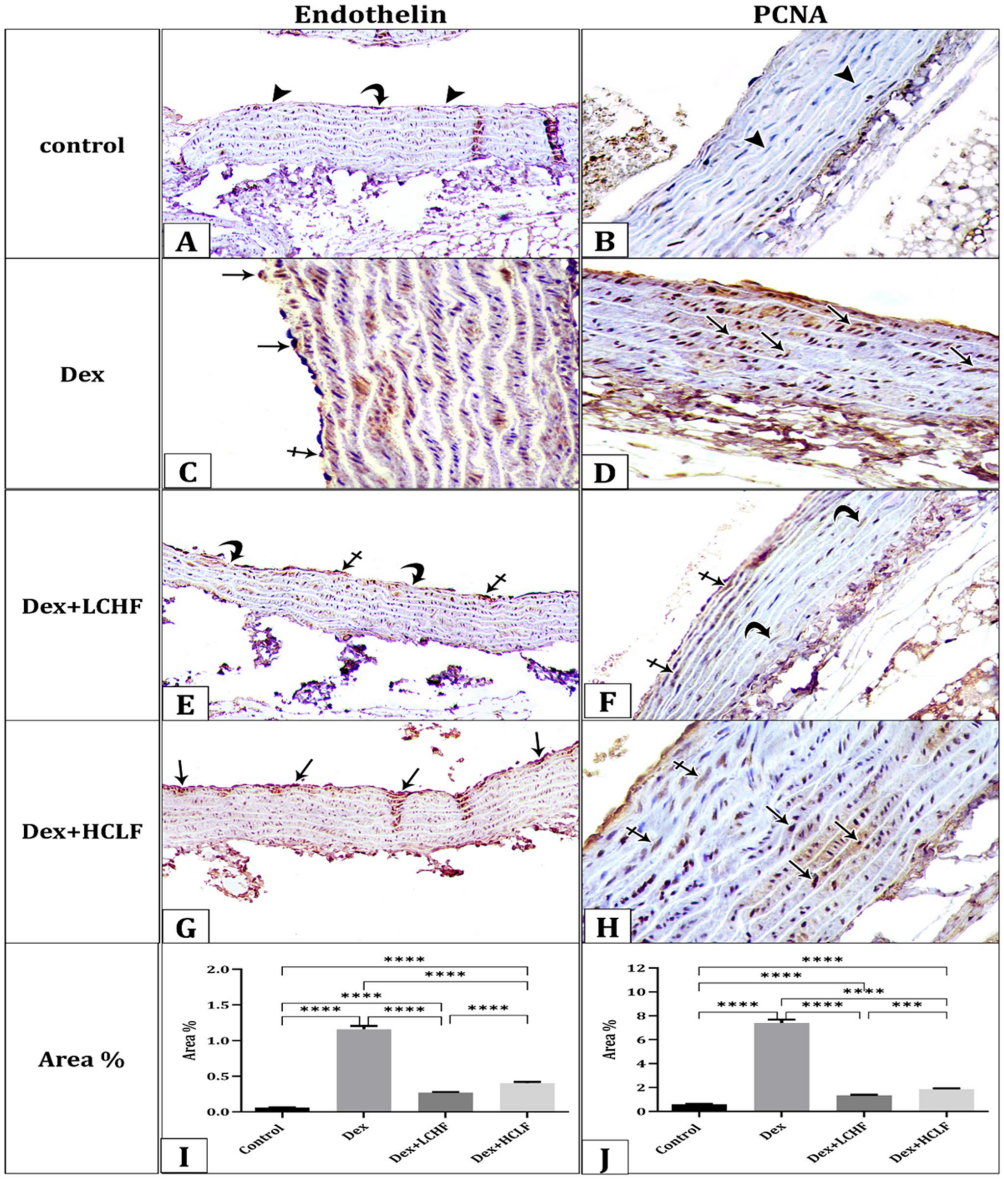
A photomicrograph of sections of the aorta immunohistochemically stained with Endothelin and PCNA. (A). The control group shows negative (arrowheads) to weak (curved arrow) endothelin expression in the cytoplasm of the endothelial cells and (B). Negative PCNA expression (arrowhead) in the nuclei of the smooth muscle fibers of the tunica media. (C) DEX group showing strong dark brown (arrows) expression of Endothelin (C) and PCNA (D). DEX + LCHF group shows weak (curved arrows) to moderate (crossed arrows) expression of Endothelin (E) and PCNA (F). DEX+ HCLF group showing moderate (crossed arrows) to strong dark brown (arrows) expression of Endothelin (G) and PCNA (H). (I,J) represent the mean area % of Endothelin and PCNA expression. Data were expressed as mean ± SEM. Statistically significant if *p ≤ 0.05: **p < 0.01: ***p < 0.001: ****p < 0.0001.

Immunohistochemical staining for Endothelin and PCNA showed varied expressions among the groups. The control group had negative to weak endothelin and negative PCNA-immune reactions observed in the cytoplasm of the endothelial cells and the nuclei of the smooth muscle fibers of the tunica media, respectively (Figures 2A,B). The DEX group demonstrated strong dark brown endothelin and PCNA-immune reactions in the cytoplasm of the endothelial cells and the nuclei of the smooth muscle fibers of the tunica media, respectively (Figures 2C,D). Conversely, the DEX + LCHF group showed weak to moderate expressions, suggesting partial protection from DEX-induced effects (Figures 2E,F). The DEX + HCLF group exhibited moderate to strong expressions, reflecting ongoing cellular stress and pathology (Figures 2G,H). The mean area % of endothelin and PCNA reaction in all groups was presented (Figures 2I,J). There was a significant increase in endothelin and PCNA reaction in the DEX and DEX + HCLF groups compared with the control group, whereas insignificant increases in the DEX + LCHF group compared with the control.

**Figure 3:**
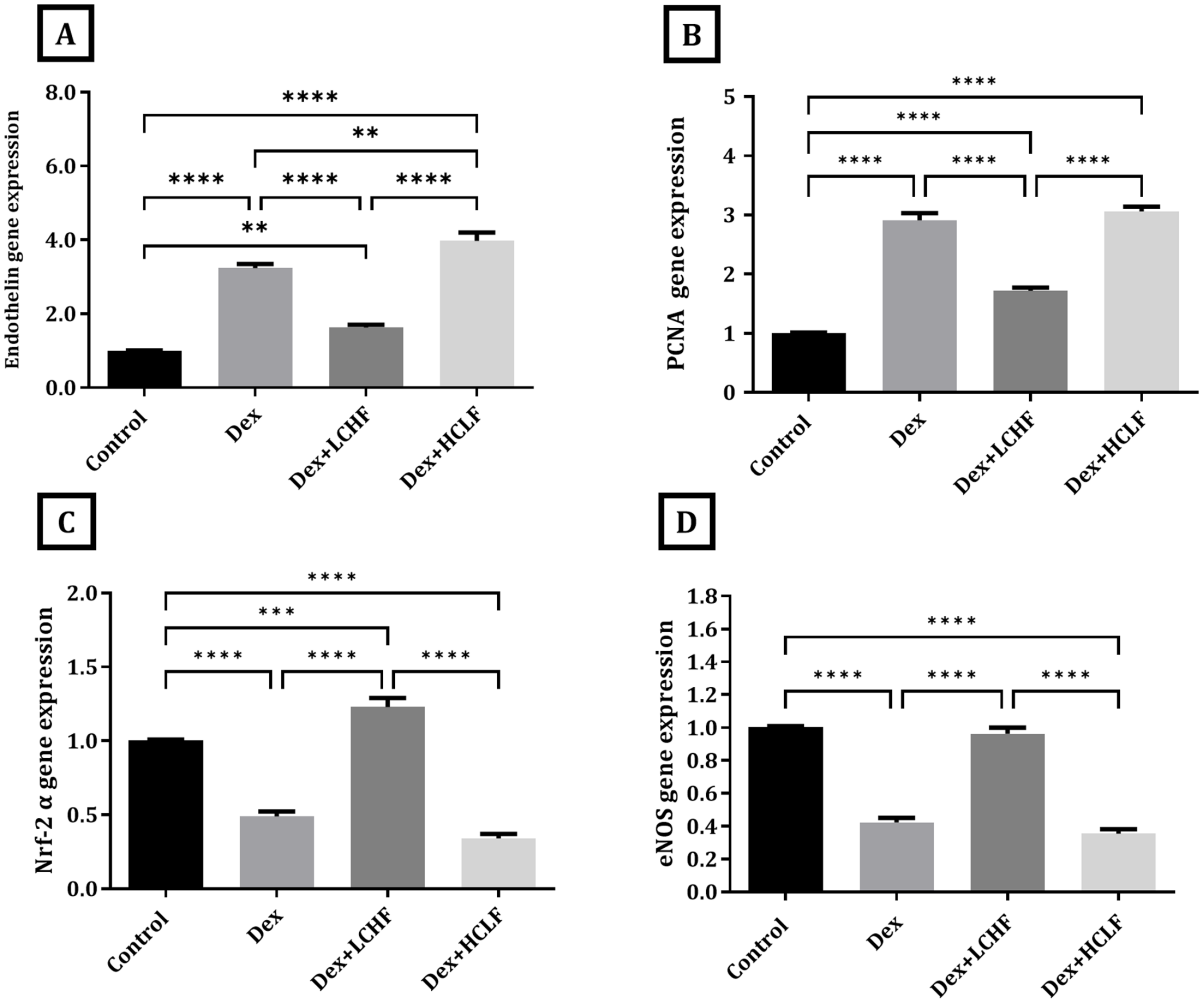
LCHF and HCLF impact on the gene expression of endothelial dysfunction markers (A) Endothelin gene expression; (B) PCNA gene expression; and (C) Nrf-2*α* gene expression. (D) eNOS gene expression. Y axis of gene expression (Fold expression relative to control). Data were expressed as mean ± SEM. DEX, DEX + LCHF, and DEX + HCLF groups. Statistically significant if *p ≤ 0.05: **p < 0.01: ***p < 0.001: ****p < 0.0001.

The molecular gene expression further confirmed pathological changes indicative of endothelial dysfunction and oxidative stress. Dex + LCHF showed a downregulation of the expression of Endothelin and PCNA compared to the DEX group (*p* < 0.0001). In contrast, the DEX + HCLF group had similar or higher expression of PCNA and Endothelin, respectively, compared to the DEX group (Figures 3A,B). In contrast, the DEX + LCHF group showed an upregulation of Nuclear factor erythroid 2-related factor 2 *α* (NrF-2α) and endothelial nitric oxide synthase (eNOS) (*p* < 0.0001), while the DEX + HCLF group had similar or lower expression compared to DEX group (Figures 3C,D).

**Figure 4:**
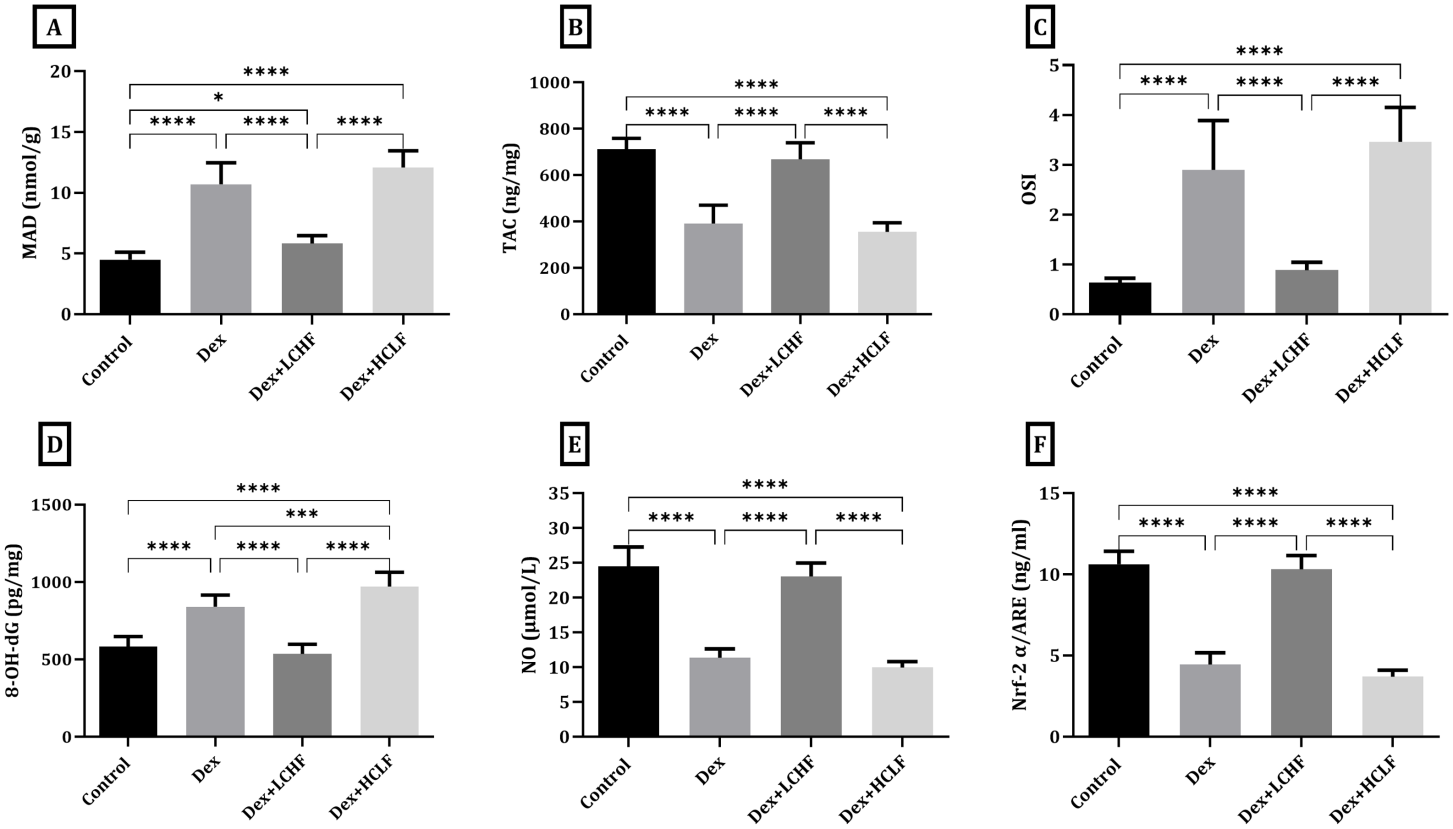
LCHF and HCLF impact on oxidative stress markers in aortic tissue homogenate. (A) Tissue Malondialdehyde (MDA); (B) total Antioxidant Capacity (TAC); (C) Oxidative stress Index (D) Tissue 8-OH-dG. (E) Tissue Nitric Oxide (NO). (F) Tissue Nrf-2 α/ARE. Data were expressed as mean ± SEM. DEX, DEX + LCHF, and DEX + HCLF groups. Statistically significant if *p ≤ 0.05: **p < 0.01: ***p < 0.001: ****p < 0.0001.

Measuring tissue homogenate level of oxidative stress markers showed that the DEX+ LCHF group had a decrease in the tissue malondialdehyde (MAD), 8-hydroxy-2′-deoxyguanosine (8-OH-dG), and oxidative stress index (OSI) levels (*p* < 0.0001) while the level of total antioxidant capacity (TAC), NO, and NRF-2α was increased in comparison to DEX group (*p* < 0.0001) (Figure 4). The HCLF diet failed to confer similar protective effects, showing values similar to those of the DEX group.

### 3.3 Effect of LCHF diet on autophagy and ER stress

**Figure 5:**
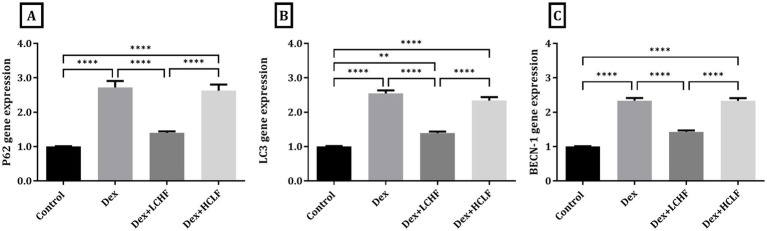
LCHF and HCLF diets impact on Autophagy markers. (A) p62 gene expression; (B) LC3 gene expression; and (C) BECN-1 gene expression. Y axis of gene expression (Fold expression relative to control). Data were expressed as mean ± SEM. DEX, DEX + LCHF, and DEX + HCLF groups. Statistically significant if *p ≤ 0.05: **p < 0.01: ***p < 0.001: ****p < 0.0001.

**Figure 6:**
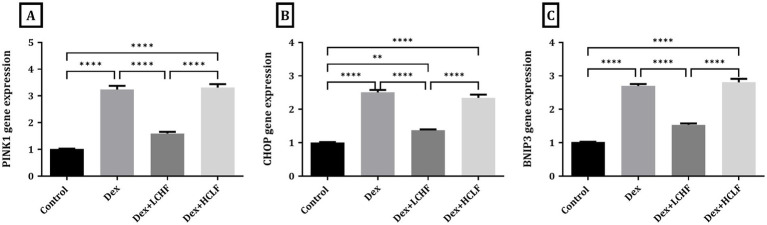
LCHF and HCLF diets impact on ER stress markers. (A) PINK1 gene expression; (B) CHOP gene expression; and (C) BNIP3 gene expression. Y axis of gene expression (Fold expression relative to control). Data were expressed as mean ± SEM. DEX, DEX + LCHF, and DEX + HCLF groups. Statistically significant if *p ≤ 0.05: **p < 0.01: ***p < 0.001: ****p < 0.0001.

DEX treatment disrupted autophagy, shown by increased expression of the p62, LC3, and BECN-1 genes. DEX + LCHF showed almost normal levels of these gene expressions, with a significant decrease compared to the DEX group (*p* < 0.0001), indicating enhanced cellular homeostasis (Figure 5). Similarly, ER stress markers were normalized in the DEX + LCHF group compared to increased gene expression in the DEX and DEX + HCLF groups (p < 0.0001 for PINK1, CHOP, and BNIP3) (Figure 6).

**Figure 7:**
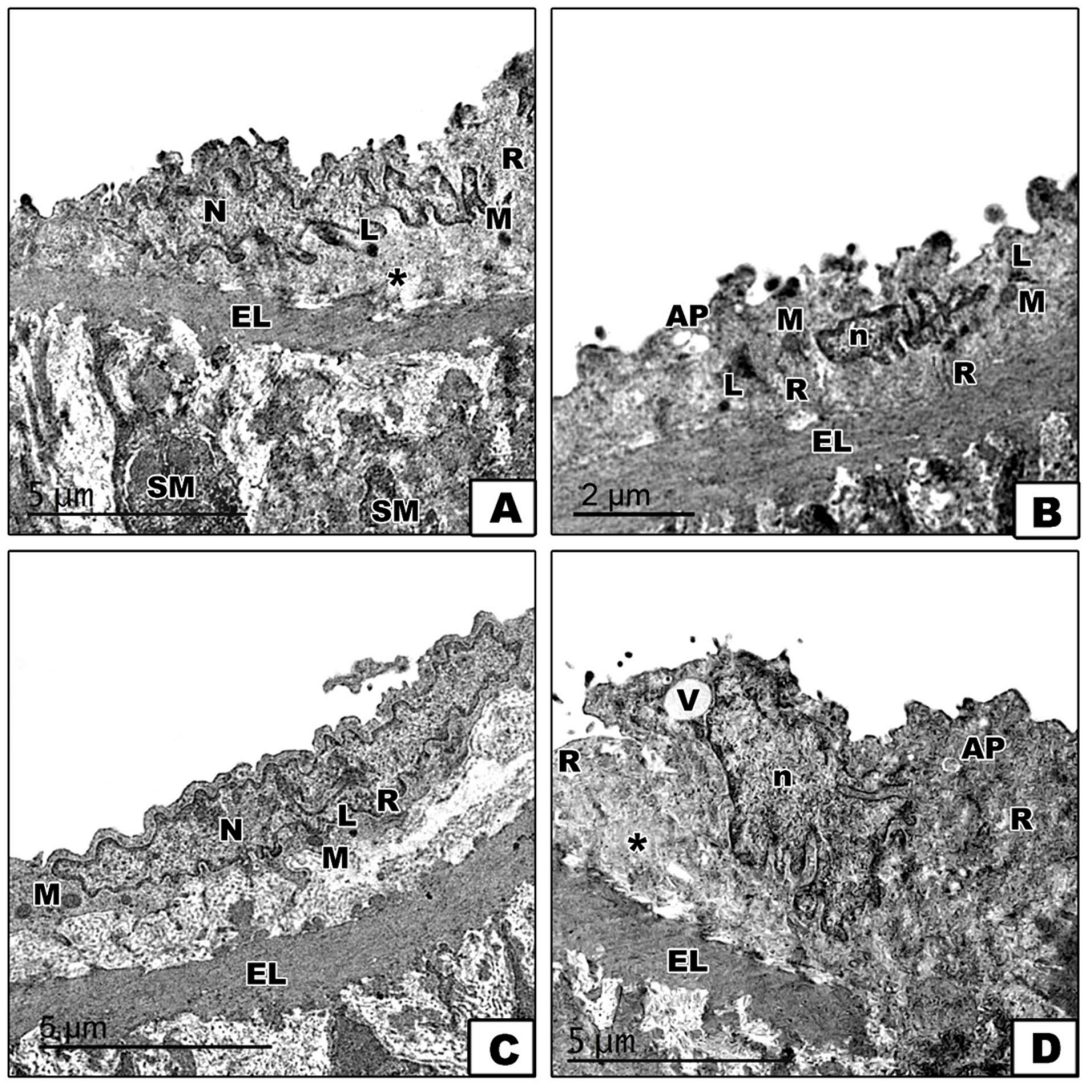
Electron micrographs of sections in the aorta. (A). In the control group, the endothelial cell of the tunica intima appears with an euchromatic nucleus (N), and its cytoplasm contains mitochondria (M), rough endoplasmic reticulum (R), and a few lysosomes (L). The subendothelial connective tissue layer shows collagen fibers (*). The internal elastic lamina (EL) separates the endothelial cell from the tunica media’s smooth muscle (SM) fibers. (B). In the DEX-treated group, the endothelial cell has a shrunken heterochromatic nucleus (n), and its cytoplasm shows numerous lysosomes (L), autophagosome (AP), dilated rough endoplasmic reticulum (R) with fewer attached ribosomes and mitochondria (M). (C). In the DEX + LCHF treated group, the endothelial cell appears nearly similar to the control group with a euchromatic nucleus (N), and its cytoplasm contains mitochondria (M), rough endoplasmic reticulum (R) and few lysosomes (L). (D). In the DEX + HCLF treated group, the endothelial cell has a shrunken nucleus (n), appears partially separated from the subendothelial connective tissue layer (*) and its cytoplasm shows vaccules (V) autophagosome (AP) and dilated rough endoplasmic reticulum (R) with fewer attached ribosomes. Notice the presence of the interna elastic lamina (EL) between the tunica intima and the tunica media.

To further confirm the impact of diets on ER stress and autophagy, the electron microscopy study of the aorta revealed varying ultrastructural changes across the four groups. In the control group, the aorta displayed a normal ultrastructure with endothelial cells characterized by euchromatic nuclei and a cytoplasm containing rough endoplasmic reticulum, mitochondria, and a few lysosomes, all separated from the tunica media’s smooth muscle fibers by the internal elastic lamina, with the subendothelial connective layer housing collagen fibers (Figure 7A). Conversely, the DEX group presented endothelial cells with shrunken heterochromatic nuclei, a cytoplasm with numerous lysosomes, autophagosomes, and dilated rough endoplasmic reticulum with fewer attached ribosomes, together which indicates cellular stress, including ER stress and autophagic activity (Figure 7B). The DEX + LCHF group appeared to mitigate these changes, with endothelial cells showing an ultrastructure nearly identical to the control group, including euchromatic nuclei and a cytoplasm with normal rough endoplasmic reticulum, a few lysosomes, and mitochondria (Figure 7C). On the other hand, DEX + HCLF endothelial cells exhibited shrunken nuclei. They were partially separated from the subendothelial connective tissue layer, with autophagosomes, dilated rough endoplasmic reticulum with fewer attached ribosomes, and vacuoles in the cytoplasm (Figure 7D).

**Figure 8:**
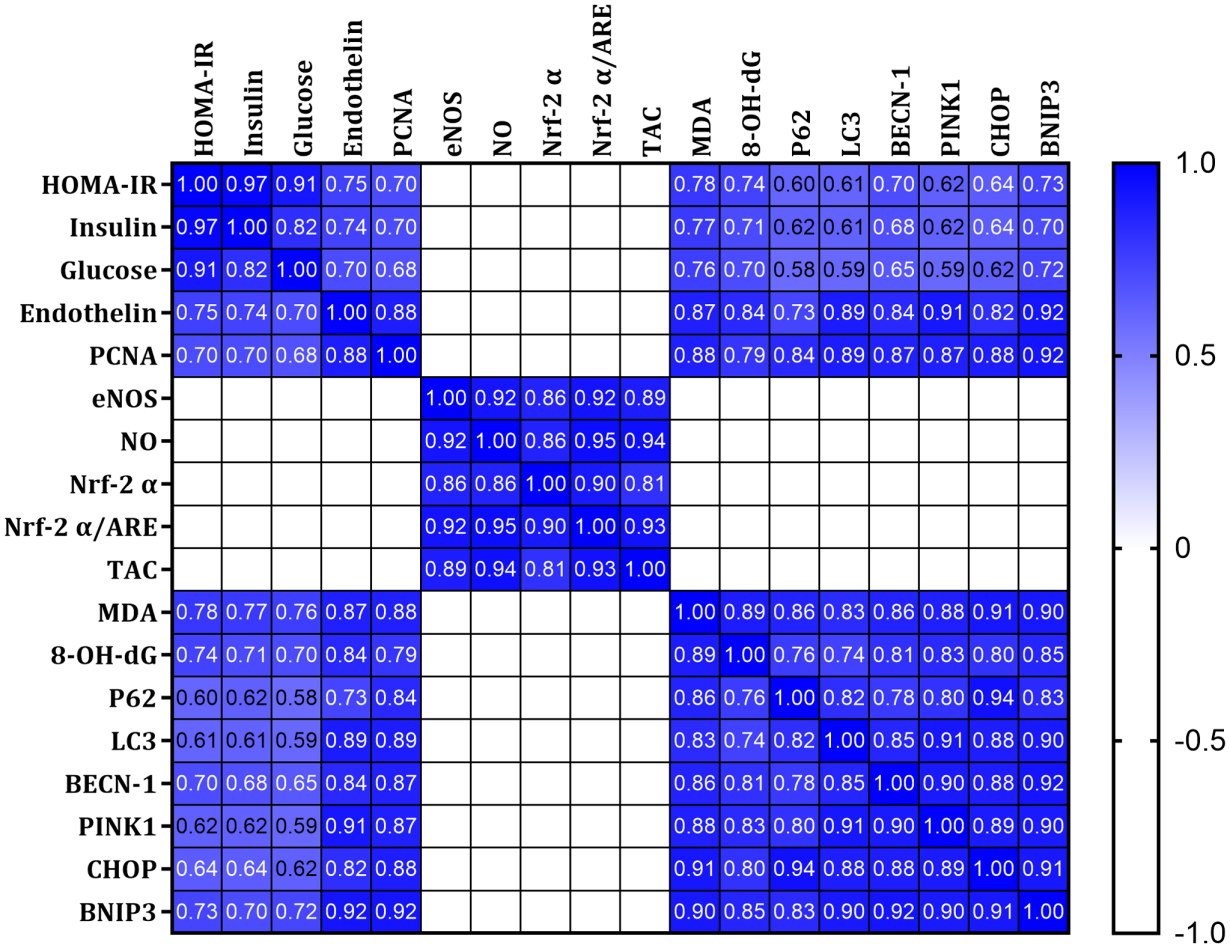
Pearson’s correlations of all measured and calculated data in all studied rats. Blue indicates a strong positive correlation, and white suggests a strong negative correlation.

Pearson’s coefficient r was measured for all parameters, including HOMA-IR, fasting plasma insulin, and glucose. A strong positive correlation was found between HOMA-IR, insulin, glucose, Endothelin, PCNA, MDA,8-OH-dG, p62, LC3, PINK1, BECN-1 CHOP, and BNIP3 (Figure 8 Blue). In contrast, a strong negative correlation was found with eNOS, NO, Nrf-2α, and TAC (Figure 8 white). This indicates a relationship between impaired insulin signaling and dysregulated glucose metabolism with ER stress pathways and autophagy.

## 4 Discussion

Endothelial dysfunction and insulin resistance, including insulin resistance-related morbidities such as metabolic syndrome, diabetes mellitus Type II (T2DM), and obesity, are linked in a complex and reciprocal manner. Therefore, insulin resistance is associated with increased susceptibility of patients to atherosclerotic-related cardiovascular and cerebrovascular complications, such as cerebral stroke and coronary artery disease (26, 27). Using the DEX-induced metabolic syndrome model, we confirm the link between insulin resistance and endothelial dysfunction and show the impact of LCHF in preventing endothelial dysfunction with its associated ER stress and autophagy.

The primary explanation for the link between insulin resistance and vascular endothelial dysfunction is impaired insulin action due to receptor resistance and its consequent hyperinsulinemia. Under normal conditions, insulin activates downstream signaling through insulin receptor (IR) substrate 1/2 (IRS-1/2) and phosphatidylinositol 3-kinase (PI3K) in endothelial cells. This leads to the activation of endothelial nitric oxide synthase (eNOS), resulting in nitric oxide (NO) production and vasodilation (28). Further, insulin stimulates the Ras/mitogen-activated protein kinase (MAPK) signaling pathway, enhancing the vasoconstrictor peptide endothelin-1 (ET-1) expression. In insulin resistance, these physiological actions of insulin are impaired. Increased vascular tone stemming from ET-1 is associated with sympathetic vasoconstriction, which counteracts NO-mediated vasodilation during hyperinsulinemia.

Furthermore, reducing insulin-induced dilation and blood flow are characteristic features of insulin resistance-related morbidities such as T2DM and obesity (29). Another mechanism is the shedding of the insulin receptors (IR) from the endothelial surface, which leads to compromised overall insulin signaling pathways, such as impairment of IRS-PI3K-Akt insulin pathway by TRAF3IP2-mediated activation of JNK and IKKβ, in addition to overactivation of the MAPK-ET-1 pathway (30). The endothelial dysfunction in the insulin resistance state is exacerbated by a decrease in blood and oxygen supply, leading to a defense mechanism overexpression of endothelial cell PCNA (31, 32), which was prominent in the current study.

Oxidative stress is crucial in developing endothelial dysfunction in insulin resistance (33, 34). This study confirmed this role by showing increased MDA, 8-OH-dG, and OSI levels and decreased TAC, NO, and Nrf-2*α* levels. Additionally, eNOS and Nrf-2α mRNA expression was downregulated in the DEX-induced metabolic syndrome group compared to the control group.

Oxidative stress leads to oxidative alterations in the arterial wall, disrupting intracellular redox homeostasis and causing cellular damage and endothelial cell dysfunction (35). The pathophysiological mechanisms by which oxidative stress induces endothelial dysfunction include reducing eNOS expression and de-creasing NO bioavailability (36). This reduction leads to exaggerated vasoconstriction and induces proinflammatory and thrombotic status (37). ROS-induced inflammation also contributes to endothelial dysfunction by activating NF-κB which translocated to the nucleus and activates its target genes involved in endothelial dysfunction, such as cell adhesion molecules (VCAM-1 and ICAM-1) and inflammatory mediators like IL-6 and TNF-α Moreover, ROS-induced mitochondrial dysfunction of endothelial cells is an important mechanism. Excessive ROS can harm the mitochondria and lead to mitochondrial damage and dysfunction, accelerating ROS-induced endothelial dysfunction (38, 39). Recently, it has been reported that endothelial cell senescence induced by oxidized low-density lipoprotein (ox-LDL) plays a crucial role as a mediator of endothelial damage in metabolic syndrome-related oxidative stress. ROS in ox-LDL-of endothelial cells increases the production of senescence-associated *β*-galactosidase (SA-β-gal) with concomitant downregulating CREB/Nrf2 antioxidant signaling with induction of cell senescence (40). Furthermore, endothelial cell apoptosis is markedly enhanced by oxidative stress (41, 42).

Autophagy is an essential mechanism for supporting cellular homeostasis due to its ability to degrade and remove misfolded proteins, damaged organelles, and pathogens (43). Autophagy is a defense mechanism against cellular stresses, such as nutrient deficiencies, caloric restriction, and oxidative stress injury. Thus, it acts as a stress response that promotes cell survival in unfavorable conditions (44). Therefore, autophagy dysregulation is associated with pathological conditions, including metabolic disorders (45), notably insulin resistance (46). Excessive nutrient consumption associated with oxidative stress can lead to mitochondrial dysfunctions that enhance mitophagy for selective clearance of the excess damaged mitochondria. This mechanism is vital in the pathophysiology of insulin resistance, as it helps maintain cellular energy balance and reduce oxidative damage, which is crucial for preserving insulin sensitivity (47).

In the current study, we identified an exaggerated expression of autophagy molecular markers p62, LC3, and BECN-1 mRNA in the aortic tissue sample of the metabolic syndrome group compared to the control group in addition to the appearance of autophagosomes by electron microscopic examination. These findings confirmed the cellular stress of aortic tissues in the metabolic syndrome group induced by insulin resistance and the above-discussed associated oxidative stress.

The microtubule-associated protein LC3 is induced by lipid accumulation in the insulin resistance state and consequently stimulates the progression of the autophagy process (44). LC3 is a receptor for adaptor protein p62/sequestosome 1 (SQSTM1), also known as p62, an interaction that promotes autophagosome formation. The autophagy pathway is completed by the fusion of lysosomes and autophagosomes, leading to the formation of autophagolysosomes and the degradation and recycling of their aggregated contents. Following this, p62 is degraded in autophagolysosomes, and consequently, its level is used to assess the efficiency and completion of the autophagy process (48). Similarly, the upregulation of BECN1 acts as a defense mechanism in insulin resistance. It has been reported that hyperactive BECN1 can activate autophagy in response to cellular stress caused by insulin resistance, helping to alleviate the pathology, enhance systemic insulin sensitivity, and regulate energy metabolism (49). The current findings regarding dysregulated autophagy supported those published before and stated that autophagy impairment contributes to vascular dysfunction and atherosclerosis pathogenesis by increasing cell death in aortic tissue (50).

Oxidative stress is known to be linked to autophagy stimulation and ER stress induction (51). Mitochondrial oxidative stress affects the transmission of redox signals through the mitochondrial-associated ER membrane. Consequently, mitochondria and ER activate the autophagic pathways to relieve oxidative stress damage (52, 53).

The current study showed similar results, where DEX treatment increased the expression of ER stress markers (PINK1, CHOP, and BNIP3). Additionally, electron microscopy of aortic tissue revealed a dilated rough endoplasmic reticulum with fewer ribosomes and mitochondria. These findings align with studies showing increased PINK1, CHOP, and BNIP3 biomarkers and ER stress in the insulin-resistance state (54–58). It also showed the relation between ER stress and insulin resistance-related morbidities such as T2DM (59), obesity and lipotoxicity (60), non-alcoholic fatty liver disease (61), Alzheimer’s Disease (62), and polycystic ovary syndrome (63). It is also reported that prolonged ER stress promotes endothelial insulin resistance development and progression, demonstrating the reciprocal connection between both (64).

Investigating the potential protective role of the LCHF diet against insulin resistance-associated oxidative stress, autophagy dysregulation, and ER stress and their consequent aortic endothelial dysfunction was the primary objective of this study, bringing novelty to the current work. In the current study, we investigated the potential protective effect of the LCHF diet against the endothelial dysfunction of metabolic syndrome at the histological, molecular, and biochemical levels.

LCHF, or the Ketogenic diet (KD), is characterized by insufficient glucose for body energy demands. Lipids serve as substrates for the hepatic production of ketone bodies (KB), especially *β* hydroxybutyrate (BHB), becoming the primary energy substrates. In addition, KBs can act as signaling molecules, employing multiple molecular and metabolic effects. These effects make KD an effective therapy for various diseases, including obesity, diabetes, and some cancers (65). Interestingly, the LCHF diet has been used recently in a clinical trial with marked improvement of some endothelial dysfunction markers (66).

The endothelium is the first-line tissue exposed to circulating KB. BHB has anti-inflammatory and antioxidative effects, which could help in understanding the role of BHB on the physiology of endothelial cell function and integrity. This fact supports the concept that BHB, at low concentrations (nutritional ketosis level), helps endothelial and vascular function in metabolic disease compared to KB levels observed in diabetic ketoacidosis, which contributes to the development of insulin-resistance-induced vasculopathy (67).

Low-dose KB also induces a shift from small and dense low-density lipoprotein to large and buoyant low-density lipoprotein. This shift is associated with lowering cardiovascular disease risk and protecting the vascular endothelium from the harmful effects of dyslipidemia (68).

Our findings indicate that the LCHF diet enhances epithelial integrity and protects against endothelial dysfunction. This is shown by the downregulation of Endothelin and PCNA and an increase in NO expression compared to the DEX group. Moreover, the LCHF protects the endothelium from insulin resistance-induced oxidative stress by preventing an increase in oxidative stress markers and significantly boosting total antioxidant capacity compared to the DEX group.

This result is supported by previous studies showing that an LCHF ketogenic diet induces nutritional ketosis, which decreases oxidative stress [54], although the exact mechanism remains elusive. Some mechanisms that explain the antioxidant effect of the LCHF include that βHB acts as a scavenger for hydroxyl radicals (•OH) (69) and upregulation of NRF-2 that modulates the expression of various genes involved in the antioxidant response such as glutamate-cysteine ligase catalytic subunit (GCLC), glutathione peroxidase, superoxide dismutase, catalase, and heme oxygenase (70) Our findings on NRF-2α and eNOS gene expressions further support its role in protecting against insulin resistance-related endothelial dysfunction induced by oxidative stress (71–73).

In the current study, the LCHF group of animals significantly displayed a lower expression of autophagy molecular markers p62, LC3, and BECN-1 mRNA in the aortic tissue samples compared to the DEX group, with levels like those of the control group. This was further confirmed by electron microscopy. These findings indicate enhanced cellular homeostasis due to the LCHF diet.

Autophagy is induced in response to increased cellular energetic demands by nutrient deprivation (autophagy inducer) in peripheral tissues and the brain (74). The KD mimics the biochemical actions of fasting-induced nutrient deprivation; thus, LCHF feeding has cellular responses parallel to those induced by intermittent energy restriction (75). KD restores autophagic activity, as evidenced by the downregulation of p62, LC3, and BECN-1 gene expression (76, 77), as detected in the current study. The downregulation of these autophagy markers observed in our study indicates marked release of the aortic tissue endothelial cell from the stressor induced by the insulin resistance effect and preservation of endothelial cell homeostasis to nearly normal metabolic adaptation by the LCHF ketogenic diet.

The discrepancy between the lower autophagy effect observed with LCHF in our study and the expected increase of autophagy by KB may be due to various conditions. The complex interplay between the diet’s metabolic effects and DEX’s hormonal modulation could result in altered cellular responses. For example, in Dex-induced stress, cells may respond differently to the LCHF compared to a non-stressed state, leading to alterations in autophagy expression. In addition, different tissues or cell types may respond differently to the metabolic and stress conditions induced by DEX and LCHF. Autophagy is also a dynamic process that can be regulated at different stages and is influenced highly by nutritional and energy status and duration, all of which can differ in various experimental conditions. Future studies could further investigate these pathways for a more comprehensive understanding of these complex interactions.

No significant difference exists between the LCHF and control groups, suggesting that the LCHF diet effectively regulates cellular homeostasis. It prevents excessive autophagy, which could harm endothelial cell function. This balanced autophagy may help maintain normal vascular remodeling in the arteries, indicating its protective role.

Korovila et al., 2021 observed downregulation of autophagy gene expression, notably, light chain 3B (LC3-I/LC3-II), Atg5, Atg5-Atg12 complex, and p62 in the high-fat diet; however, their experiment investigated liver autophagy rather than blood vessels, as in our study (78). Similar to the current research, Liśkiewicz et al. (75) discussed the role of the LCHF diet by linking KD to improved cellular metabolism and mitochondrial biogenesis and protection against oxidative stress by modulating autophagy; though, their study was carried out on hippocampal and cerebrocortical autophagy (75).

Regarding the role of autophagy in vascular remodeling, autophagy has a dual effect on the vascular wall. It can employ protective and damaging effects on the vascular wall layers, including intima, media and adventitia, and the vascular endothelial cells. Thus, autophagy upregulation could influence vascular remodeling and insulin resistance-induced vascular diseases (79).

In addition, Guo et al. (80) reported a decreased expression of the p62 gene with increased autophagic vacuoles during vascular remodeling in metabolic diseases. They explained that it induces endothelium-dependent vasodilation, NO bioavailability, and reduces endothelial senescence. This p62 downregulation is crucial in preventing endothelial dysfunction (80).

In the LCHF-fed group, we found that the ER stress markers for PINK1, CHOP, and BNIP3 gene expression normalized compared to the exacerbated conditions in the DEX and DEX + HCLF groups. By electron microscopic examination, the rough endo-plasmic reticulum appeared within normal morphology with few lysosomes. Also, the size and shape of the mitochondria were normal. These findings confirm our hypothesis that the LCHF ketogenic diet alleviates ER endoplasmic reticulum stress, which has been supported recently (81–83).

Montiel et al. (81) correlated ER stress and autophagy in the pathogenesis of insulin resistance-related comorbidities. They stated that autophagy is directly linked to the ER-produced (UPR), which is activated to restore cellular homeostasis and prevent pathological proteotoxicity (81, 84). The UPR is prompted by a low cellular energy state (85). The LCHF diet can cut this vicious cycle by providing the body with sufficient energy derived from the oxidation of KB ketone bodies. Consequently, it protects against ER stress (86).

Reduced carbohydrate intake in the LCHF diet leads to decreased demand for insulin, providing *β*-cells with transient rest and improving proinsulin processing within β-cells. This decreases protein processing activity in the ER, protecting it from the development of ER stress (87).

The antioxidant effect of the LCHF KD plays a role in preventing endothelial cells’ ER stress. It has been reported that ROS generated from mitochondrial fission can activate NLRP3 inflammasome, exacerbating ER stress and further impairing mitochondrial morphology and function (88). Thus, the antioxidant effect of the BHB produced during the LCHF diet aborts this ROS-induced ER stress (89).

To confirm the impact of the nonpharmacologic dietary intervention of the LCHF diet, we investigated the dietary intervention of high-carbohydrates low-fat (HCLF) on a parallel group of rats with isocaloric values. The current study found that the HCLF diet significantly deteriorated all examined markers compared to the LCHF-fed animal. Oxidative stress, exaggerated autophagy, and endoplasmic reticulum stress markers were worse and reflected more exaggerated deterioration of endothelial dysfunction markers. HCLF-induced hyperinsulinemia could explain these effects (90).

The current findings underscore the important role of oxidative stress, autophagy dysregulation, and ER stress in the pathophysiology of endothelial dysfunction, which is a hallmark of several metabolic and cardiovascular diseases. For instance, insulin resistance, as seen in metabolic syndrome, exacerbates these cellular stress pathways, contributing to vascular damage. This study demonstrated the protective effects of the LCHF diet, signifying its potential therapeutic value for preventing endothelial dysfunction and for mitigating cardiovascular risks associated with metabolic syndrome, atherosclerosis, and possibly Type 2 diabetes. Translational studies could explore the application of the LCHF diet in these specific patient populations. These insights would provide a deeper mechanistic understanding and guide the development of targeted dietary interventions in clinical settings.

## 5 Conclusion

The interplay between oxidative stress, autophagy dysregulation, and ER stress is essential in the pathogenesis of insulin resistance-induced endothelial dysfunction in the large arteries, notably the aorta. An LCHF diet protects against the insulin resistance state and its related comorbidities, including endothelial dysfunctions. The current study confirmed the role of the LCHF diet as an antioxidant, cellular autophagy modifier, and ER stress modulator at the biochemical, molecular, and histological levels with subsequent protection against endothelial dysfunction. Our study thus recommends using the LCHF ketogenic diet as a nonpharmacological dietary intervention for prophylactic and therapeutic management of insulin resistance-induced endothelial dysfunction.

## Data Availability

The original contributions presented in the study are included in the article/Supplementary material, further inquiries can be directed to the corresponding author.

## References

[1] Lee S-H Park S-Y Choi CS. Insulin resistance: from mechanisms to therapeutic strategies. Diabetes Metab J. (2022) 46:15–37. doi: 10.4093/dmj.2021.0280, PMID: 34965646 PMC8831809

[2] Petersen MC Shulman GI. Mechanisms of insulin action and insulin resistance. Physiol Rev. (2018) 98:2133–223. doi: 10.1152/physrev.00063.2017, PMID: 30067154 PMC6170977

[3] Cahill PA Redmond EM. Vascular endothelium - gatekeeper of vessel health. Atherosclerosis. (2016) 248:97–109. doi: 10.1016/j.atherosclerosis.2016.03.007, PMID: 26994427 PMC6478391

[4] Godo S Shimokawa H. Endothelial functions. Arterioscler Thromb Vasc Biol. (2017) 37:e108–14. doi: 10.1161/ATVBAHA.117.309813 28835487

[5] Bavani NG Saneei P Hassanzadeh Keshteli A Yazdannik A Falahi E Sadeghi O. Magnesium intake, insulin resistance and markers of endothelial function among women. Public Health Nutr. (2021) 24:5777–85. doi: 10.1017/S1368980021001063, PMID: 33719988 PMC10195414

[6] Chen C Zhang B Xue J Li Z Dou S Chen H. Pathogenic role of endoplasmic reticulum stress in diabetic corneal endothelial dysfunction. Invest Ophthalmol Vis Sci. (2022) 63:4. doi: 10.1167/iovs.63.3.4, PMID: 35238867 PMC8899864

[7] Osman A Benameur T Korashy HM Zeidan A Agouni A. Interplay between endoplasmic reticulum stress and large extracellular vesicles (microparticles) in endothelial cell dysfunction. Biomedicines. (2020) 8:409. doi: 10.3390/biomedicines8100409, PMID: 33053883 PMC7599704

[8] Maamoun H Abdelsalam SS Zeidan A Korashy HM Agouni A. Endoplasmic reticulum stress: a critical molecular driver of endothelial dysfunction and cardiovascular disturbances associated with diabetes. Int J Mol Sci. (2019) 20:1658. doi: 10.3390/ijms20071658, PMID: 30987118 PMC6480154

[9] Ogata M Hino S-i Saito A Morikawa K Kondo S Kanemoto S. Autophagy is activated for cell survival after endoplasmic reticulum stress. Mol Cell Biol. (2006) 26:9220–31. doi: 10.1128/MCB.01453-06, PMID: 17030611 PMC1698520

[10] Hosoi T Nomura J Tanaka K Ozawa K Nishi A Nomura Y. Link between endoplasmic reticulum stress and autophagy in neurodegenerative diseases. Endopl. Retic. Stress Dis. (2017) 4:4. doi: 10.1515/ersc-2017-0004

[11] Yang J Yao S. Jnk-Bcl-2/Bcl-xl-Bax/Bak pathway mediates the crosstalk between Matrine-induced autophagy and apoptosis via interplay with Beclin 1. Int J Mol Sci. (2015) 16:25744–58. doi: 10.3390/ijms161025744, PMID: 26516844 PMC4632824

[12] Keane KN Cruzat VF Carlessi R de Bittencourt PIH Newsholme P. Molecular events linking oxidative stress and inflammation to insulin resistance and Β-cell dysfunction. Oxidative Med Cell Longev. (2015) 2015:1–15. doi: 10.1155/2015/181643, PMID: 26257839 PMC4516838

[13] Okada T Yoshida H Akazawa R Negishi M Mori K. Distinct roles of activating transcription factor 6 (Atf6) and double-stranded Rna-activated protein kinase-like endoplasmic reticulum kinase (perk) in transcription during the mammalian unfolded protein response. Biochem J. (2002) 366:585–94. doi: 10.1042/BJ20020391, PMID: 12014989 PMC1222788

[14] Seckold R Fisher E de Bock M King BR Smart CE. The ups and downs of low-carbohydrate diets in the Management of Type 1 diabetes: a review of clinical outcomes. Diabet Med. (2018) 36:326–34. doi: 10.1111/dme.13845 30362180

[15] Park SB Yang SJ. Ketogenic diet preserves muscle mass and strength in a mouse model of type 2 diabetes. PLoS One. (2024) 19:6651. doi: 10.1371/journal.pone.0296651, PMID: 38198459 PMC10781088

[16] Alnami A Bima A Alamoudi A Eldakhakhny B Sakr H Elsamanoudy A. Modulation of dyslipidemia markers Apo B/Apo a and triglycerides/Hdl-cholesterol ratios by low-carbohydrate high-fat diet in a rat model of metabolic syndrome. Nutrients. (2022) 14:1903. doi: 10.3390/nu14091903, PMID: 35565871 PMC9102123

[17] Bima A Eldakhakhny B Alamoudi AA Awan Z Alnami A Abo-Elkhair SM. Molecular study of the protective effect of a low-carbohydrate, high-fat diet against brain insulin resistance in an animal model of metabolic syndrome. Brain Sci. (2023) 13:1383. doi: 10.3390/brainsci13101383, PMID: 37891752 PMC10605073

[18] Nemmar A Al-Salam S Beegam S Yuvaraju P Ali BH. Aortic oxidative stress, inflammation and DNA damage following pulmonary exposure to cerium oxide nanoparticles in a rat model of vascular injury. Biomol Ther. (2019) 9:376. doi: 10.3390/biom9080376, PMID: 31426470 PMC6722935

[19] Bancroft JD Layton C. The hematoxylins and eosin. Bancroft's theory and practice of histological techniques. Amsterdam: Elsevier (2019). 126–138.

[20] Münzel T Daiber A Steven S Tran LP Ullmann E Kossmann S. Effects of noise on vascular function, oxidative stress, and inflammation: mechanistic insight from studies in mice. Eur Heart J. (2017) 38:2838–49. doi: 10.1093/eurheartj/ehx081, PMID: 28329261 PMC5837459

[21] Liu J Liu Z Hu X Zhang Y Zhang S. Synthetic E-selectin prevents postoperative vascular restenosis by inhibiting nuclear factor Κb in rats. Mol Med Rep. (2018) 17:5065–73. doi: 10.3892/mmr.2018.8536 29393453 PMC5865970

[22] Jackson P Blythe D. Immunohistochemical techniques. Bancroft's theory and practice of histological techniques Amsterdam: Elsevier (2013). p. 381–426.

[23] Khalaf HA Elsamanoudy AZ Abo-Elkhair SM Hassan FE Mohie PM Ghoneim FM. Endoplasmic reticulum stress and mitochondrial injury are critical molecular drivers of Alcl(3)-induced testicular and Epididymal distortion and dysfunction: protective role of taurine. Histochem Cell Biol. (2022) 158:97–121. doi: 10.1007/s00418-022-02111-2, PMID: 35511291 PMC9247002

[24] Klisic A Kavaric N Stanisic V Vujcic S Spasojevic-Kalimanovska V Ninic A. Endocan and a novel score for dyslipidemia, oxidative stress and inflammation (Doi score) are independently correlated with glycated hemoglobin (Hba(1c)) in patients with prediabetes and type 2 diabetes. Arch Med Sci. (2019) 16:42–50. doi: 10.5114/aoms.2019.87541, PMID: 32051704 PMC6963142

[25] Bima AI Mahdi AS Al Fayez FF Khawaja TM Abo El-Khair SM Elsamanoudy AZ. Cellular senescence and vitamin D deficiency play a role in the pathogenesis of obesity-associated subclinical atherosclerosis: study of the potential protective role of vitamin D supplementation. Cells. (2021) 10:920. doi: 10.3390/cells10040920, PMID: 33923622 PMC8073712

[26] Gómez-Hernández A de Las HN López-Pastor AR García-Gómez G Infante-Menéndez J González-López P. Severe hepatic insulin resistance induces vascular dysfunction: improvement by liver-specific insulin receptor isoform a gene therapy in a murine diabetic model. Cells. (2021) 10:2035. doi: 10.3390/cells10082035, PMID: 34440804 PMC8392327

[27] Sánchez DCV Castellanos SG Sandoval MEV García AG. B-cell activating factor increases related to adiposity, insulin resistance, and endothelial dysfunction in overweight and obese subjects. Life. (2022) 12:634. doi: 10.3390/life12050634, PMID: 35629302 PMC9146198

[28] Mather KJ Steinberg HO Baron AD. Insulin resistance in the vasculature. J Clin Invest. (2013) 123:1003–4. doi: 10.1172/JCI67166, PMID: 23454764 PMC3582147

[29] Limberg JK Soares RN Padilla J. Role of the autonomic nervous system in the hemodynamic response to hyperinsulinemia-implications for obesity and insulin resistance. Curr Diab Rep. (2022) 22:169–75. doi: 10.1007/s11892-022-01456-1, PMID: 35247145 PMC9012695

[30] Padilla J Manrique-Acevedo C Martinez-Lemus LA. New insights into mechanisms of endothelial insulin resistance in type 2 diabetes. Am J Physiol Heart Circ Physiol. (2022) 323:H1231–8. doi: 10.1152/ajpheart.00537.2022, PMID: 36331555 PMC9705017

[31] Sui D-X Zhou H-M Wang F Zhong M Zhang W Ti Y. Cell death-inducing Dff45-like effector C gene silencing alleviates pulmonary vascular remodeling in a type 2 diabetic rat model. J Diabetes Investig. (2018) 9:741–52. doi: 10.1111/jdi.12768, PMID: 29078040 PMC6031506

[32] Wu Z Geng J Qi Y Li J Bai Y Guo Z. Mir-193-3p attenuates the vascular remodeling in pulmonary arterial hypertension by targeting Pak4. Pulm Circ. (2020) 10:4919. doi: 10.1177/2045894020974919, PMID: 33354317 PMC7734527

[33] Shaito A Aramouni K Assaf R Parenti A Orekhov A Yazbi AE. Oxidative stress-induced endothelial dysfunction in cardiovascular diseases. Front Biosci. (2022) 27:0105. doi: 10.31083/j.fbl2703105 35345337

[34] Zhang Y Li J-J Xu R Wang X-P Zhao X-Y Fang Y. Nogo-B mediates endothelial oxidative stress and inflammation to promote coronary atherosclerosis in pressure-overloaded mouse hearts. Redox Biol. (2023) 68:102944. doi: 10.1016/j.redox.2023.102944, PMID: 37890359 PMC10633694

[35] Taniyama Y Griendling KK. Reactive oxygen species in the vasculature. Hypertension. (2003) 42:1075–81. doi: 10.1161/01.hyp.0000100443.09293.4f 14581295

[36] Levine AB Punihaole D Levine TB. Characterization of the role of nitric oxide and its clinical applications. Cardiology. (2012) 122:55–68. doi: 10.1159/000338150 22722323

[37] Santillo M Colantuoni A Mondola P Guida B Damiano S. Nox signaling in molecular cardiovascular mechanisms involved in the blood pressure homeostasis. Front Physiol. (2015) 6:194. doi: 10.3389/fphys.2015.00194, PMID: 26217233 PMC4493385

[38] Davidson SM Duchen MR. Endothelial mitochondria. Circ Res. (2007) 100:1128–41. doi: 10.1161/01.res.0000261970.18328.1d 17463328

[39] Muste JC Russell MW Chen AX Seth K Iyer AI Valentim CCS. Functional imaging of mitochondria in age-related macular degeneration using Flavoprotein fluorescence. Ophthal Surg Lasers Imaging Retina. (2023) 54:24–31. doi: 10.3928/23258160-20221214-03 36626211

[40] Zhao Y Li Y Li H Shi S. Dopamine D1 receptor activation ameliorates ox-Ldl-induced endothelial cell senescence via Creb/Nrf2 pathway. Exp Cell Res. (2023) 425:113542. doi: 10.1016/j.yexcr.2023.113542, PMID: 36894051

[41] Luo S Wang F Chen S Chen A Wang Z Gao X. Nrp2 promotes atherosclerosis by upregulating Parp1 expression and enhancing low shear stress-induced endothelial cell apoptosis. FASEB J. (2022) 36:e22079. doi: 10.1096/fj.202101250rr, PMID: 35028975

[42] Zhao K Han D He S-R Wu L-Y Liu W-Y Zhong Z-M. N-acetyl-L-cysteine attenuates oxidative stress-induced bone marrow endothelial cells apoptosis by inhibiting Bax/caspase 3 pathway. Biochem Biophys Res Commun. (2023) 656:115–21. doi: 10.1016/j.bbrc.2023.03.045, PMID: 36963348

[43] Gallagher LE Chan EYW. Early Signalling events of autophagy. Essays Biochem. (2013) 55:1–15. doi: 10.1042/bse0550001, PMID: 24070467

[44] Galluzzi L Green DR. Autophagy-independent functions of the autophagy machinery. Cell. (2019) 177:1682–99. doi: 10.1016/j.cell.2019.05.026 31199916 PMC7173070

[45] Xie W Zhou J. Aberrant regulation of autophagy in mammalian diseases. Biol Lett. (2018) 14:20170540. doi: 10.1098/rsbl.2017.0540, PMID: 29321247 PMC5803588

[46] Sadeghi A Niknam M Momeni-Moghaddam MA Shabani M Aria H Bastin A. Crosstalk between autophagy and insulin resistance: evidence from different tissues. Eur J Med Res. (2023) 28:456. doi: 10.1186/s40001-023-01424-9, PMID: 37876013 PMC10599071

[47] Tsilingiris D Tzeravini E Koliaki C Dalamaga M Kokkinos A. The role of mitochondrial adaptation and metabolic flexibility in the pathophysiology of obesity and insulin resistance: an updated overview. Curr Obes Rep. (2021) 10:191–213. doi: 10.1007/s13679-021-00434-0, PMID: 33840072

[48] Yoshii SR Mizushima N. Monitoring and measuring autophagy. Int J Mol Sci. (2017) 18:1865. doi: 10.3390/ijms18091865, PMID: 28846632 PMC5618514

[49] Kuramoto K He C. The secretory function of Becn1 in metabolic regulation. Autophagy. (2021) 17:3262–3. doi: 10.1080/15548627.2021.1953849, PMID: 34281478 PMC8525985

[50] Madrigal-Matute J de Bruijn J van Kuijk K Riascos-Bernal DF Diaz A Tasset I. Protective role of chaperone-mediated autophagy against atherosclerosis. Proc Natl Acad Sci USA. (2022) 119:e2121133119. doi: 10.1073/pnas.2121133119 35363568 PMC9168839

[51] Liu X Hussain R Mehmood K Tang Z Zhang H Li Y. Mitochondrial-endoplasmic reticulum communication-mediated oxidative stress and autophagy. Biomed Res Int. (2022) 2022:6459585:1–12. doi: 10.1155/2022/6459585 36164446 PMC9509228

[52] Levine B Kroemer G. Autophagy in the pathogenesis of disease. Cell. (2008) 132:27–42. doi: 10.1016/j.cell.2007.12.018, PMID: 18191218 PMC2696814

[53] Lan B He Y Sun H Zheng X Gao Y Li N. The roles of mitochondria-associated membranes in mitochondrial quality control under endoplasmic reticulum stress. Life Sci. (2019) 231:116587. doi: 10.1016/j.lfs.2019.116587, PMID: 31220526

[54] Wang K Zhou Z Huang L Kan Q Wang Z Wu W. Pink1 dominated mitochondria associated genes signature predicts abdominal aortic aneurysm with metabolic syndrome. Biochim Biophys Acta (BBA) - Mol Basis Dis. (2024) 1870:166919. doi: 10.1016/j.bbadis.2023.166919, PMID: 38251428

[55] Jiang Y Wang Z Ma B Fan L Yi N Lu B. Glp-1 improves adipocyte insulin sensitivity following induction of endoplasmic reticulum stress. Front Pharmacol. (2018) 9:1168. doi: 10.3389/fphar.2018.01168, PMID: 30459598 PMC6232689

[56] Choi JW Jo A Kim M Park HS Chung SS Kang S. Bnip3 is essential for mitochondrial bioenergetics during adipocyte Remodelling in mice. Diabetologia. (2015) 59:571–81. doi: 10.1007/s00125-015-3836-9, PMID: 26693709

[57] Ramos-Lopez O Riezu-Boj JI Milagro FI Moreno-Aliaga MJ Martinez JA. Endoplasmic reticulum stress epigenetics is related to adiposity, dyslipidemia, and insulin resistance. Adipocytes. (2018) 7:137–42. doi: 10.1080/21623945.2018.1447731, PMID: 29570038 PMC6152516

[58] Lemmer IL Willemsen N Hilal N Bartelt A. A guide to understanding endoplasmic reticulum stress in metabolic disorders. Mol Metab. (2021) 47:101169. doi: 10.1016/j.molmet.2021.101169, PMID: 33484951 PMC7887651

[59] Guerrero-Hernández A Leon-Aparicio D Chavez-Reyes J Olivares-Reyes JA DeJesus S. Endoplasmic reticulum stress in insulin resistance and diabetes. Cell Calcium. (2014) 56:311–22. doi: 10.1016/j.ceca.2014.08.006 25239386

[60] Yazıcı D Sezer H. *Insulin resistance, obesity and lipotoxicity*. *Obesity and Lipotoxicity*. Cham: Springer International Publishing (2017). 277–304. 10.1007/978-3-319-48382-5_12 28585204

[61] Lebeaupin C Vallée D Hazari Y Hetz C Chevet E Bailly-Maitre B. Endoplasmic reticulum stress Signalling and the pathogenesis of non-alcoholic fatty liver disease. J Hepatol. (2018) 69:927–47. doi: 10.1016/j.jhep.2018.06.008 29940269

[62] Burillo J Marqués P Jiménez B González-Blanco C Benito M Guillén C. Insulin resistance and diabetes mellitus in Alzheimer's disease. Cells. (2021) 10:1236. doi: 10.3390/cells10051236, PMID: 34069890 PMC8157600

[63] Wang C Zhang Y. Endoplasmic reticulum stress: a new research direction for polycystic ovary syndrome? DNA Cell Biol. (2022) 41:356–67. doi: 10.1089/dna.2021.1050, PMID: 35353637

[64] Li X Li Q Wu L Wang Y. Nebivolol alleviates vascular endothelial insulin resistance by inhibiting endoplasmic reticulum stress. Int Heart J. (2023) 64:283–93. doi: 10.1536/ihj.22-484, PMID: 36927931

[65] Pirola L Ciesielski O Balcerczyk A. Fat not so bad? The role of ketone bodies and ketogenic diet in the treatment of endothelial dysfunction and hypertension. Biochem Pharmacol. (2022) 206:115346. doi: 10.1016/j.bcp.2022.115346 36384215

[66] Sánchez E Santos M-D Nuñez-Garcia M Bueno M Sajoux I Yeramian A. Randomized clinical trial to evaluate the morphological changes in the adventitial vasa Vasorum density and biological markers of endothelial dysfunction in subjects with moderate obesity undergoing a very low-calorie ketogenic diet. Nutrients. (2021) 14:33. doi: 10.3390/nu14010033, PMID: 35010908 PMC8746664

[67] Nasser S Vialichka V Biesiekierska M Balcerczyk A Pirola L. Effects of ketogenic diet and ketone bodies on the cardiovascular system: concentration matters. World J Diabetes. (2020) 11:584–95. doi: 10.4239/wjd.v11.i12.584, PMID: 33384766 PMC7754168

[68] Westman EC Yancy WS Olsen MK Dudley T Guyton JR. Effect of a low-carbohydrate, ketogenic diet program compared to a low-fat diet on fasting lipoprotein subclasses. Int J Cardiol. (2006) 110:212–6. doi: 10.1016/j.ijcard.2005.08.034 16297472

[69] Haces ML Hernández-Fonseca K Medina-Campos ON Montiel T Pedraza-Chaverri J Massieu L. Antioxidant capacity contributes to protection of ketone bodies against oxidative damage induced during hypoglycemic conditions. Exp Neurol. (2008) 211:85–96. doi: 10.1016/j.expneurol.2007.12.029, PMID: 18339375

[70] Tebay LE Robertson H Durant ST Vitale SR Penning TM Dinkova-Kostova AT. Mechanisms of activation of the transcription factor Nrf2 by redox stressors, nutrient cues, and energy status and the pathways through which it attenuates degenerative disease. Free Radic Biol Med. (2015) 88:108–46. doi: 10.1016/j.freeradbiomed.2015.06.021, PMID: 26122708 PMC4659505

[71] Malekmohammad K Sewell RDE Rafieian-Kopaei M. Antioxidants and atherosclerosis: mechanistic aspects. Biomolecules. (2019) 9:301. doi: 10.3390/biom9080301, PMID: 31349600 PMC6722928

[72] Wang C Sun Y Liu W Liu Y Afzal S Grover J. Protective effect of the curcumin-Baicalein combination against macrovascular changes in diabetic Angiopathy. Front Endocrinol. (2022) 13:953305. doi: 10.3389/fendo.2022.953305 PMC9433877 36060932

[73] Dauth A Bręborowicz A Ruan Y Tang Q Zadeh JK Böhm EW. Sulodexide prevents hyperglycemia-induced endothelial dysfunction and oxidative stress in porcine retinal arterioles. Antioxidants. (2023) 12:388. doi: 10.3390/antiox12020388, PMID: 36829947 PMC9952154

[74] Bagherniya M Butler AE Barreto GE Sahebkar A. The effect of fasting or calorie restriction on autophagy induction: a review of the literature. Ageing Res Rev. (2018) 47:183–97. doi: 10.1016/j.arr.2018.08.004 30172870

[75] Liśkiewicz D Liśkiewicz A Nowacka-Chmielewska MM Grabowski M Pondel N Grabowska K. Differential response of hippocampal and Cerebrocortical autophagy and ketone body metabolism to the ketogenic diet. Front Cell Neurosci. (2021) 15:733607. doi: 10.3389/fncel.2021.733607, PMID: 34456688 PMC8385303

[76] Jiang T Harder B Rojo de la Vega M Wong PK Chapman E Zhang DD. P62 links autophagy and Nrf2 signaling. Free Radic Biol Med. (2015) 88:199–204. doi: 10.1016/j.freeradbiomed.2015.06.014, PMID: 26117325 PMC4628872

[77] Kong C Yan X Liu Y Huang L Zhu Y He J. Ketogenic diet alleviates colitis by reduction of colonic group 3 innate lymphoid cells through altering gut microbiome. Signal Transduct Target Ther. (2021) 6:154. doi: 10.1038/s41392-021-00549-9, PMID: 33888680 PMC8062677

[78] Korovila I Höhn A Jung T Grune T Ott C. Reduced liver autophagy in high-fat diet induced liver steatosis in New Zealand obese mice. Antioxidants. (2021) 10:501. doi: 10.3390/antiox10040501, PMID: 33804819 PMC8063826

[79] Ren H Dai R Nik Nabil WN Xi Z Wang F Xu H. Unveiling the dual role of autophagy in vascular Remodelling and its related diseases. Biomed. Pharmacother. (2023) 168:115643. doi: 10.1016/j.biopha.2023.115643 37839111

[80] Guo L Zhang B Zhang W Xie Y Chen X Sun X. Inhibition of carbohydrate metabolism potentiated by the therapeutic effects of oxidative phosphorylation inhibitors in Colon Cancer cells. Cancers. (2024) 16:1399. doi: 10.3390/cancers16071399, PMID: 38611076 PMC11010912

[81] Montiel T Gómora-García JC Gerónimo-Olvera C Heras-Romero Y Bernal-Vicente BN Pérez-Martínez X. Modulation of the autophagy-lysosomal pathway and endoplasmic reticulum stress by ketone bodies in experimental models of stroke. J Neurochem. (2023) 166:87–106. doi: 10.1111/jnc.15852, PMID: 37328918

[82] Ma Q Jiang L You Y Ni H Ma L Lin X. Ketogenic diet ameliorates high-fat diet-induced insulin resistance in mouse skeletal muscle by alleviating endoplasmic reticulum stress. Biochem Biophys Res Commun. (2024) 702:149559. doi: 10.1016/j.bbrc.2024.149559 38341923

[83] Qiao Q Tian S Zhang Y Che L Li Q Qu Z. A ketogenic diet may improve cognitive function in rats with temporal lobe epilepsy by regulating endoplasmic reticulum stress and synaptic plasticity. Mol Neurobiol. (2023) 61:2249–64. doi: 10.1007/s12035-023-03659-3, PMID: 37870676

[84] Niu M Dai X Zou W Yu X Teng W Chen Q. Autophagy, endoplasmic reticulum stress and the unfolded protein response in intracerebral hemorrhage. Transl Neurosci. (2017) 8:37–48. doi: 10.1515/tnsci-2017-0008, PMID: 28729917 PMC5444040

[85] Balchin D Hayer-Hartl M Hartl FU. In vivo aspects of protein folding and quality control. Science. (2016) 353:4354. doi: 10.1126/science.aac4354, PMID: 27365453

[86] Makievskaya CI Popkov VA Andrianova NV Liao X Zorov DB Plotnikov EY. Ketogenic diet and ketone bodies against ischemic injury: targets, mechanisms, and therapeutic potential. Int J Mol Sci. (2023) 24:2576. doi: 10.3390/ijms24032576, PMID: 36768899 PMC9916612

[87] Myette-Côté É Durrer C Neudorf H Bammert TD Botezelli JD Johnson JD. The effect of a short-term low-carbohydrate, high-fat diet with or without Postmeal walks on glycemic control and inflammation in type 2 diabetes: a randomized trial. Am J Physiol Regul Integr Comp Physiol. (2018) 315:R1210–9. doi: 10.1152/ajpregu.00240.2018, PMID: 30303707 PMC6734060

[88] Minutoli L Puzzolo D Rinaldi M Irrera N Marini H Arcoraci V. Ros-mediated Nlrp3 Inflammasome activation in brain, heart, kidney, and testis ischemia/reperfusion injury. Oxidative Med Cell Longev. (2016) 2016:2183026. doi: 10.1155/2016/2183026, PMID: 27127546 PMC4835650

[89] Gómora-García JC Montiel T Hüttenrauch M Salcido-Gómez A García-Velázquez L Ramiro-Cortés Y. Effect of the ketone body, D-Β-Hydroxybutyrate, on Sirtuin2-mediated regulation of mitochondrial quality control and the autophagy-lysosomal pathway. Cells. (2023) 12:486. doi: 10.3390/cells12030486, PMID: 36766827 PMC9914182

[90] Joseph A Parvathy S Varma KK. Hyperinsulinemia induced altered insulin signaling pathway in muscle of high fat- and carbohydrate-fed rats: effect of exercise. J Diabetes Res. (2021) 2021:5123241:1–10. doi: 10.1155/2021/5123241, PMID: 33708999 PMC7929694

[91] Tang S-T Su H Zhang Q Tang H-Q Wang C-J Zhou Q. Sitagliptin inhibits Endothelin-1 expression in the aortic endothelium of rats with Streptozotocin-induced diabetes by suppressing the nuclear factor-Κb/Iκbα system through the activation of amp-activated protein kinase. Int J Mol Med. (2016) 37:1558–66. doi: 10.3892/ijmm.2016.2578, PMID: 27122056 PMC4866950

[92] Ghoneim FM Alrefai H Elsamanoudy AZ Abo El-Khair SM Khalaf HA. The protective role of prenatal alpha lipoic acid supplementation against pancreatic oxidative damage in offspring of Valproic acid-treated rats: histological and molecular study. Biology. (2020) 9:239. doi: 10.3390/biology9090239, PMID: 32825436 PMC7564314

[93] Chen ZHI Fu Q Shen B Huang X Wang KUN He P. Enhanced P62 expression triggers concomitant autophagy and apoptosis in a rat chronic spinal cord compression model. Mol Med Rep. (2014) 9:2091–6. doi: 10.3892/mmr.2014.2124, PMID: 24715058

[94] Ghoneim FM Abo-Elkhair SM Elsamanoudy AZ Shabaan DA. Evaluation of endothelial dysfunction and autophagy in fibromyalgia-related vascular and cerebral cortical changes and the ameliorative effect of Fisetin. Cells. (2022) 11:48. doi: 10.3390/cells11010048, PMID: 35011610 PMC8750434

[95] Zhang D Zheng N Fu X Shi J Zhang J. Dl-3-N-butylphthalide attenuates myocardial ischemia reperfusion injury by suppressing oxidative stress and regulating cardiac Mitophagy via the Pink1/Parkin pathway in rats. J Thorac Dis. (2022) 14:1651–62. doi: 10.21037/jtd-22-585, PMID: 35693588 PMC9186216

[96] Liang X Yang Y Huang Z Zhou J Ye L Zhong X. Panax Notoginseng Saponins mitigate cisplatin induced nephrotoxicity by inducing Mitophagy via Hif-1α. Oncotarget. (2017) 8:102989–3003. doi: 10.18632/oncotarget.19900, PMID: 29262539 PMC5732705

[97] Nishimura J Dewa Y Muguruma M Kuroiwa Y Yasuno H Shima T. Effect of Fenofibrate on oxidative DNA damage and on gene expression related to cell proliferation and apoptosis in rats. Toxicol Sci. (2007) 97:44–54. doi: 10.1093/toxsci/kfm011, PMID: 17264098

[98] Abo El-khair SM Ghoneim FM Shabaan DA Elsamanoudy AZ. Molecular and ultrastructure study of endoplasmic reticulum stress in hepatic steatosis: role of hepatocyte nuclear factor 4α and inflammatory mediators. Histochem Cell Biol. (2019) 153:49–62. doi: 10.1007/s00418-019-01823-2, PMID: 31637472

