# Supplementary figures and images for: The role of low-carbohydrate, high-fat diet in modulating autophagy and endoplasmic reticulum stress in aortic endothelial dysfunction of metabolic syndrome animal model
Source: Front Nutr. 2024 Nov 13;11:1467719. doi: 10.3389/fnut.2024.1467719 (PMC11603365; doi:10.3389/fnut.2024.1467719)

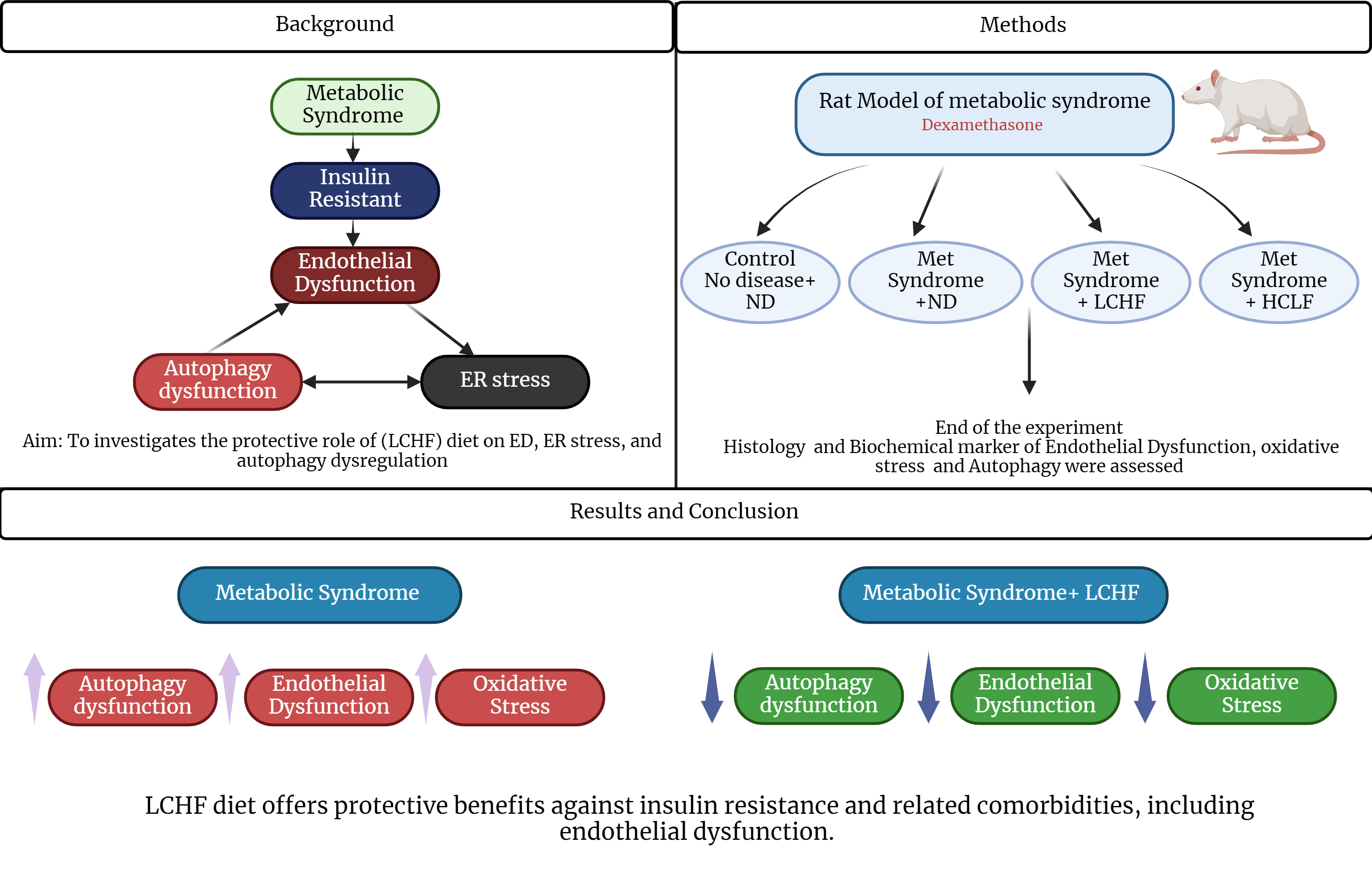

Supplement: Supplementary file 1 [file Image_1.PNG]
